# Effects of Regulatory T Cell Depletion on NK Cell Responses against *Listeria monocytogenes* in Feline Immunodeficiency Virus Infected Cats

**DOI:** 10.3390/v11110984

**Published:** 2019-10-24

**Authors:** Rita D. Simões, Alora LaVoy, Gregg A. Dean

**Affiliations:** Center for Comparative Medicine and Translational Research, College of Veterinary Medicine, North Carolina State University, Raleigh, NC 27606, USA; rdsimoes@ncsu.edu (R.D.S.); alora.lavoy@colostate.edu (A.L.)

**Keywords:** feline immunodeficiency virus, NK cells, regulatory T cells, innate immunity, Treg depletion

## Abstract

Regulatory T cells (Treg) are key players in the maintenance of peripheral tolerance, preventing autoimmune diseases and restraining chronic inflammatory diseases. Evidence suggests Treg cells and NK cells have important roles in feline immunodeficiency virus (FIV) pathogenesis; however, in vivo studies investigating the interplay between these two cell populations are lacking. We previously described innate immune defects in FIV-infected cats characterized by cytokine deficits and impaired natural killer cell (NK) and NK T cell (NKT) functions. In this study, we investigated whether in vivo Treg depletion by treatment with an anti-feline CD25 monoclonal antibody would improve the innate immune response against subcutaneous challenge with *Listeria monocytogenes* (Lm). Treg depletion resulted in an increased overall number of cells in Lm-draining lymph nodes and increased proliferation of NK and NKT cells in FIV-infected cats. Treg depletion did not normalize expression of perforin or granzyme A by NK and NKT cells, nor did Treg depletion result in improved clearance of Lm. Thus, despite the quantitative improvements in the NK and NKT cell responses to Lm, there was no functional improvement in the early control of Lm. CD1a+ dendritic cell percentages in the lymph nodes of FIV-infected cats were lower than in specific-pathogen-free control cats and failed to upregulate CD80 even when Treg were depleted. Taken together, Treg depletion failed to improve the innate immune response of FIV-infected cats against Lm and this may be due to dendritic cell dysfunction.

## 1. Introduction

Three key immunological features of lentiviral infection are the lack of an effective immune response against the virus, susceptibility to opportunistic pathogens, and generalized immune activation. The underlying mechanisms that lead to these immunologic alterations are complex and interrelated. Regulatory T cells (Treg) are thought to contribute to the control of excessive immune activation on one hand and suppression of the anti-viral response on the other hand [1]. Such Treg-mediated suppression can render the host immune system unable to clear chronic infections [2]. During chronic human, simian, or feline immunodeficiency virus infection (HIV-1, SIV, and FIV, respectively), Treg cells are capable of suppressing antiviral responses in vitro [3,4,5,6]. Our lab has shown that Treg suppression of antiviral responses is also exerted in vivo during chronic FIV infection and that transient depletion of CD25^+^ Treg cells improved the antiviral response [7]. Treg cells thus contribute to viral persistence in lentiviral infection by suppressing adaptive immune responses. The role of Treg in the suppression of the innate immune system and susceptibility to opportunistic infections has been much less studied.

We have employed the intracellular opportunistic pathogen *Listeria monocytogenes* (Lm) to probe the immune defects associated with FIV. Using this immune challenge model, we found that FIV-infected cats have an impaired innate response that fails to gain initial control of bacterial replication prior to the adaptive immune response [8]. We also showed that locally delivered interleukin 15 (IL-15), a cytokine known to activate and stimulate natural killer (NK) cells, significantly restored innate immune function as measured by Lm clearance [9]. Further investigation revealed that NK cells and NK T cells (NKT) from FIV-infected cats display heightened constitutive levels of proliferation and apoptosis, and a defective response to Lm compared to the NK/NKT cell response in specific-pathogen-free (SPF) control cats [10].

The early control mechanism of Lm does not rely on T cells; however, a T cell response is required for bacterial clearance, as demonstrated by T cell-deficient mice that are able to control but not clear Lm infection [11]. It seems that NK cell production of interferon gamma after stimulation with IL-12 and IL-18 plays an important role in Lm control [12]. Several studies have shown that NK cell-depleted mice and rats fail to control initial Lm infection, resulting in a higher bacterial burden [13,14], whereas in normal animals infected with Lm, NK cells are recruited from the blood to the spleen, liver, and/or lymph nodes, and increased NK cell activity is observed during the first days of Lm infection.

Our objective in the present study was to determine whether Treg contribute to the impaired NK cell function in FIV-infected cats, and reduced capacity to clear Lm. We hypothesized that in vivo Treg cell depletion using anti-feline CD25 monoclonal antibody prior to innate immune challenge with Lm would improve the innate immune response and the clearance of bacteria.

## 2. Materials and Methods

### 2.1. Ethics Statement

All experimental manipulations and protocols were approved by North Carolina State University Institutional Animal Care and Use Committee (protocol #09-127-B). Animals were housed and cared for in accordance with standards established in the Animal Welfare Act and Guide for the Care and Use of Laboratory Animals.

### 2.2. Animals, Viral Inoculum, and Monoclonal Antibody Administration

A total of 24 SPF female cats were purchased from Liberty Research (Waverly, NY, USA) and group housed. A group of 12 cats between 16 and 18 weeks of age were infected with 3.75 × 10^5^ cell-associated and 9.75 × 10^4^ TCID-50 cell-free FIV NCSU_1_ virus [15]. Cell-free and cell associated virus inocula were mixed immediately prior to administration and each animal received half the dose by intravenous and half by intravaginal routes. Cats were considered chronically infected after 1 year. The control group consisted of 12 age-matched female SPF cats. Mouse anti-feline CD25 (9F23) [16] and mouse anti-yellow fever antigen (YFA; CRL-1689, ATCC, Manassas, VA, USA) monoclonal antibodies (mAb) were purified and certified mycoplasma and endotoxin-free (Leinco, St. Louis, MO, USA). A total of 6 FIV-infected and 6 SPF-control cats were treated with 9 mg/Kg anti-feline CD25 mAb intraperitoneally (i.p.), and 6 FIV-infected and 6 SPF-control cats were treated with 9 mg/Kg anti-YFA mAb i.p. as an isotype control mAb (IgG2a, κ light chain).

### 2.3. Listeria monocytogenes Inoculum and Bromodeoxyuridine (BrdU) Administration

Ten days after treatment with either anti-feline CD25 or anti-YFA mAb, FIV-infected and SPF-control cats were challenged with *Listeria monocytogenes*. A total of 100 µL of 2.5 × 10^6^ cfu/mL of Lm was subcutaneously injected proximal to both the right metatarsal and metacarpal footpads. All animals were similarly injected with 100 µL of PBS proximal to both the left metatarsal and metacarpal footpads (sham-inoculated control sites). FIV-infected and SPF-control cats received 30 mg/Kg BrdU (MilliporeSigma, St. Louis, MO, USA) by intraperitoneal route on days 0, 1, and 2 post-Lm inoculation.

### 2.4. Sample Collection and Processing, and Lm Quantification

Blood for complete blood counts, leukocyte differentials, and plasma isolation was collected in Vacutainer tubes (BD, Franklin Lakes, NJ, USA) containing EDTA. Peripheral blood mononuclear cells (PBMC) were isolated as previously described [17]. Plasma was isolated by centrifugation and aliquots were stored at −80 °C. Cervical and popliteal Lm-draining lymph nodes (LN) and the contralateral control LN (sham-inoculated) were harvested at necropsy. LN were weighed, bisected, and a portion was processed into single cell suspensions for phenotypic analysis or functional assays as previously described [18]. The remaining portion was homogenized and cultured for bacterial quantification as previously described [9], with the exception that the LN homogenate was plated on brain heart infusion agar.

### 2.5. Immunophenotyping

A minimum of 1 × 10^6^ cells were labeled with the following monoclonal antibodies for flow cytometric analysis. Antibody against feline CD3 (NZM1) was used unconjugated [19]; anti-CD4 (30A) [20] was conjugated to either Pacific Blue (Invitrogen, Carlsbad, CA, USA) or R-Phycoerythrin (PE, Agilent, Santa Clara, CA, USA); anti-CD8 (3.357) [21] was conjugated to either peridinin-chlorophyll-protein complex (PerCP, Agilent) or fluorescein isothiocyanate (FITC) all using standard protocols. The aforementioned antibodies were purified from hybridoma supernatants. Anti CD80-PE (B7.1.66) was provided by Dr. Mary Tompkins of North Carolina State University [21]. Anti-CD1a (Fel.5F4) was provided by Dr. Peter Moore of the University of California at Davis [22] and conjugated to allophycocyanin (APC, Agilent). Anti-CD25 (9F23) was conjugated to FITC. Anti-CD56-APC (HCD56; BioLegend, San Diego, CA, USA), unconjugated anti-MHCII (PF8J-9B; Bio-Rad Antibodies, Hercules, CA, USA), anti-mouse IgG2a-PE/Cy7 (Southern Biotech, Birmingham, AL, USA), anti-CD11c-A488 (BU15; Bio-Rad Antibodies), and anti-CD62L-PE (SK11; BD Biosciences, San Jose, CA) were purchased. Anti-mouse IgG3 was purchased from Jackson ImmunoResearch (West Grove, PA, USA), conjugated to Pacific Orange (Invitrogen) and used for secondary detection of anti-CD3 (NZM1). AnnexinV-Pacific Blue (Invitrogen) was used according to manufacturer’s instructions. Intracellular FOXP3 staining was performed using FOXP3 staining buffers and FOXP3-APC (FJK-16s; Invitrogen) as previously described [7].

Intranuclear BrdU staining was performed with BD Biosciences BrdU staining buffers and anti-BrdU-FITC antibody (3D4) according to manufacturer’s recommendations, with Ki-67-PE (B56, BD Biosciences) staining performed at the same time. Staining was performed as previously described [7] with at least 500,000 gated events collected for each sample. Gating strategy has been described elsewhere [10]. Flow cytometric data analysis was performed using FlowJo 7.6.5 (FlowJo LLC, Ashland, OR, USA).

### 2.6. Perforin and Granzyme A Production by NK and NKT Cells

Production of perforin and granzyme A by ex-vivo NK and NKT cells was assessed using a FACS-based assay. Whole cell suspensions from LN were incubated overnight at 37 °C and 5% CO_2_ in the presence of 50 U/mL human IL-2 (obtained through the NIH AIDS Reagent Program, Division of AIDS, NIAID, NIH) in RPMI 1640 medium supplemented with 10% fetal bovine serum, penicillin-streptomycin (10 IU/mL and 10 µg/mL, respectively), GlutaMax (4 mM), sodium pyruvate (1 mM), HEPES (15 mM), and 2-mercaptoethanol (Gibco, Hercules, CA, USA). The next day, monensin (BioLegend) was added to a final concentration of 2 µM and cells were incubated for 3 hours. After incubation, cells were washed in PBS (Gibco) and surface staining was performed using anti-CD56-APC (HCD56; BioLegend) and unconjugated feline anti-CD3 (NZM1). Anti-mouse IgG3 (Jackson ImmunoResearch) conjugated to Pacific Orange (Invitrogen) was used for secondary detection of anti-CD3. Cells were resuspended in 100 µL of a 4% paraformaldehyde solution at 4 °C for 15 min. After fixation, cells were washed twice with PBS and resuspended in BD Perm/Wash Buffer for 15 min on ice with agitation. Following permeabilization, cells were washed and stained with anti-perforin conjugated to A488 (DG9, BioLegend) and anti-granzyme A-Pacific Blue (CB9; BioLegend). Samples were analyzed using a BD LSRII flow cytometer, with a minimum of 500,000 gated events collected per sample. Gating strategy has been described elsewhere [10]. Flow cytometric data analysis was performed using FlowJo 7.6.5 (FlowJo LLC).

### 2.7. Viral Parameters

Quantitative real-time one-step reverse transcriptase (RT)-PCR assays were performed on a Bio-Rad MyiQ^TM^ PCR detection system (Hercules, CA, USA). QIAamp Viral RNA Mini Kit (QIAGEN, Germantown, MD, USA) was used to extract plasma RNA. Detection of plasma viremia in RNA samples was performed using NCSU_1_ FIV gag specific primers as previously described [7]. Bio-Rad MyiQ^TM^ optical system software v2.0 was used to generate a standard curve and viral RNA copies/mL were calculated. Range of detection was between 10^1^ and 10^5^ copies/mL.

FIV proviral load from PBMC and LN cells was determined by real-time PCR using specific FIV-gag and CCR5 primers and probes as previously described [7], with a limit of detection ≤10 copies of FIV per 1 µg DNA. DNA was extracted with the DNeasy Blood and Tissue Kit (QIAGEN) and 0.5 µg DNA sample or standard was used per reaction.

### 2.8. Statistical Analysis

Data were compared by 1-way ANOVA with Tukey’s post-test or by Mann–Whitney U test. Statistics were calculated using GraphPad Prism version 8.2.0 (GraphPad Software, San Diego, CA, USA).

## 3. Results

### 3.1. The Effect of Anti-CD25 mAb Treatment on Treg, NK and NKT Cells and on Viral Burden

**Figure 1:**
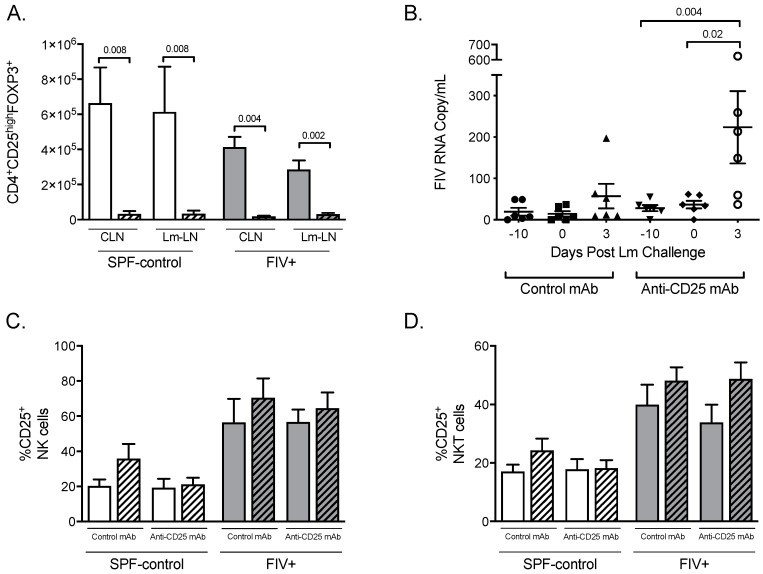
The effects of anti-CD25 mAb treatment on lymph node Treg, NK, and NKT cell numbers, and on viral burden. (**A**) CD4^+^CD25^high^FOXP3^+^ cell numbers from lymph nodes (LN) of specific-pathogen-free (SPF)-control and feline immunodeficiency virus (FIV)-infected cats treated with isotype-control monoclonal antibodies (mAb, open bars) or anti-CD25 mAb (hatched bars) are shown for control LN (CLN) and *Listeria monocytogenes* (Lm)-challenged LN (Lm-LN). (**B**) Plasma viremia was determined on days −10, 0, and 3, post-Lm challenge for FIV-infected cats receiving isotype-control mAb or anti-CD25 mAb. The percentages of natural killer (NK) cells (**C**) and NK T cells (NKT cells) (**D**) expressing CD25 in control lymph nodes (open bars) versus Lm-draining lymph nodes (hatched bars) were determined by flow cytometric analysis. Statistical analysis was performed using one-way ANOVA with Tukey’s post-test; *n* = 6 for each treatment group; *p*-values are shown above brackets that indicate group comparisons.

Twelve chronically FIV-infected cats and 12 SPF-control cats were divided into four groups of six cats and injected with either anti-CD25 mAb or isotype control antibody. In previous work, we showed that maximum Treg cell depletion occurred 10 days after anti-CD25 mAb administration [7,23]; therefore, we chose to probe the innate immune response at that time point. *Listeria monocytogenes* was injected subcutaneously next to the footpad 10 days after mAb treatment. Three days later, both the draining LN (Lm-LN) and the contralateral control LN (CLN) were removed. Expression of the forkhead transcription factor FOXP3 in CD4 T cells that also express high levels of the IL-2 receptor α-chain (CD25) is associated with the Treg suppressive phenotype in mice, humans, and cats [24,25,26]. The absolute number of CD4^+^CD25^high^FOXP3^+^ cells was reduced to between 89% and 98% in CLN and Lm-LN of both SPF-control and FIV-infected cats 3 days after Lm challenge (13 days after mAb treatment) (Figure 1A). To determine whether treatment with anti-CD25 mAb affected CD25-expressing NK and NKT cells, these populations were also assessed by flow cytometry and did not show any significant changes regardless of treatment with mAb or challenge with Lm (Figure 1C,D).

FIV plasma viremia was not altered by either anti-CD25 mAb or isotype control mAb treatment (day 0, Figure 1B). Challenge with Lm led to a six-fold increase in plasma viremia in the CD25-depleted group compared to viremia immediately prior to Lm challenge (*p* = 0.02) but not in the isotype control group (Figure 1B). Proviral load in PBMC was not altered by anti-CD25 mAb, isotype control mAb treatment, or by Lm challenge (Supplemental Figure S1A). No differences in proviral load within the CLN and Lm-LN cells were observed at 3 days post-Lm challenge between Treg cell depleted and control groups (Supplemental Figure S1B).

### 3.2. The Effect of Treg Depletion on the Quantitative Cellular Response to Lm

**Figure 2:**
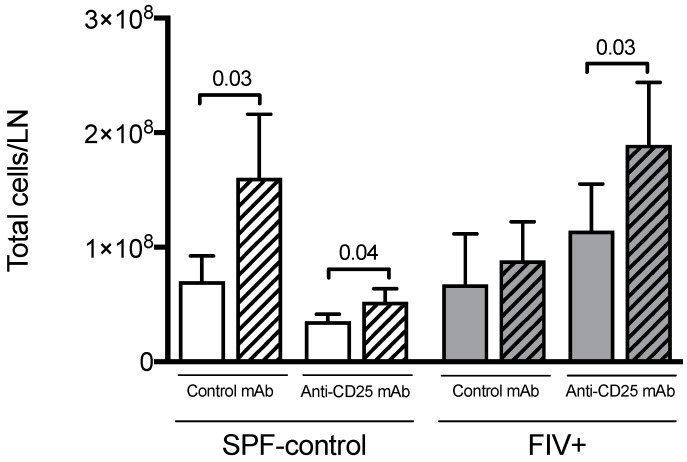
The effect of Treg depletion on total LN cellularity. The total number of cells per control lymph node (open bars) versus Lm-draining lymph node (hatched bars) was determined for SPF-control and FIV-infected cats treated with isotype control mAb or anti-CD25 mAb. Statistical analysis was performed using Mann–Whitney U test; *n* = 6 for each treatment group; *p*-values are shown above brackets that indicate group comparisons.

Previously we showed that Lm challenge resulted in an increase in weight of the draining LN of SPF-control cats but not of FIV-infected cats [8]. Here we measured the total number of cells per lymph node rather than simply comparing weight. Cellularity of LN from SPF-control animals significantly increased after challenge with Lm, whether cats were treated with isotype control mAb or anti-CD25 mAb (Figure 2). FIV-infected animals treated with isotype control mAb did not show an increased LN cellularity, mirroring previous comparisons of lymph node weight. However, Treg depletion restored the LN cellular response of FIV-infected cats upon challenge with Lm (Figure 2).

**Figure 3:**
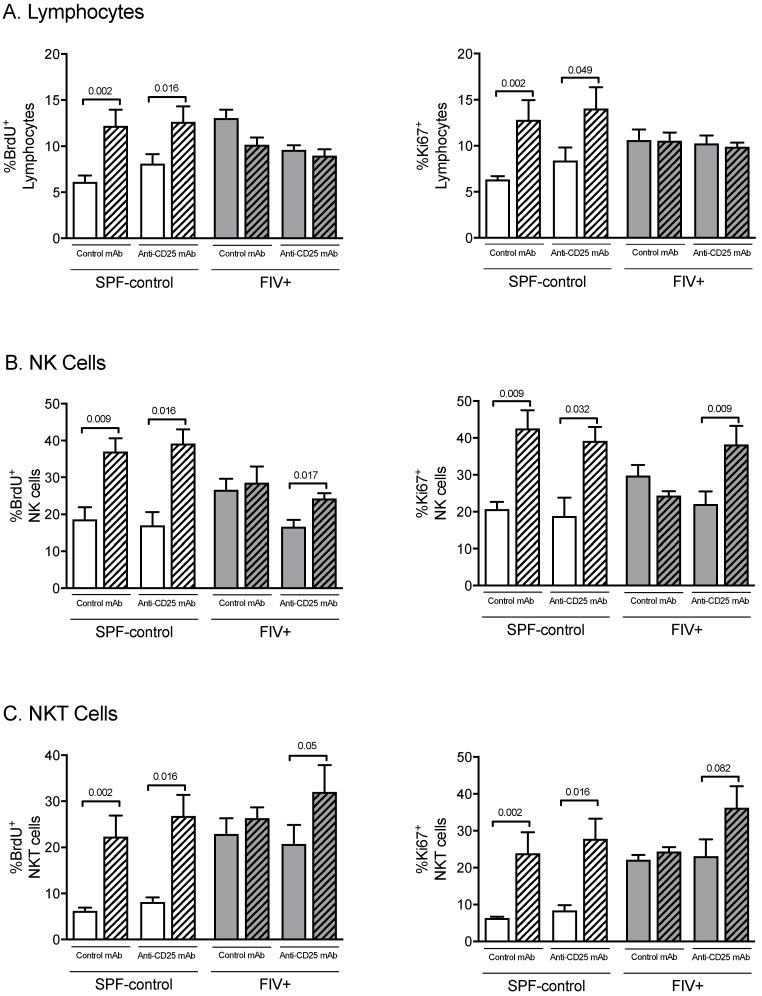
The effect of Treg depletion on NK cell and NKT cell proliferation. Cell proliferation was assessed in cats treated with isotype control mAb or anti-CD25 mAb by BrdU incorporation (left) or expression of the nuclear antigen Ki-67 (right). The percent of gated lymphocytes from the control lymph node (open bars) versus Lm-draining lymph node (hatched bars) that incorporated BrdU or expressed that Ki-67 was determined by flow cytometric analysis. (**A**) Percentage of proliferating total lymphocytes; (**B**) percentage of proliferating NK cells; (**C**) percentage of proliferating NKT cells. Statistical analysis was performed using one-way ANOVA with Tukey’s post-test; *n* = 6 for each treatment group; *p*- values are shown above brackets that indicate group comparisons.

We previously showed that after the Lm challenge, the proliferative rate of total lymphocytes and NK cells from the Lm-LN of SPF-control cats increased but no increase was observed in chronically FIV-infected cats [10]. In this study, we sought to determine if Treg cell depletion might improve the proliferative capacity in lymph nodes of FIV-infected cats in response to the Lm immune challenge. Proliferation of total lymphocytes, NK cells, and NKT cells was measured by the in vivo incorporation of BrdU and by intranuclear expression of Ki-67 in CLN and Lm-LN, 3 days after Lm challenge. Treg cell depletion increased proliferation of the lymphocyte population in SPF cats but not in FIV-infected cats (Figure 3A). Interestingly, Treg cell depletion in FIV-infected cats did result in an increased proliferation of NK cells and NKT cells in response to Lm challenge, rendering a response similar to SPF-control cats (Figure 3B,C).

**Figure 4:**
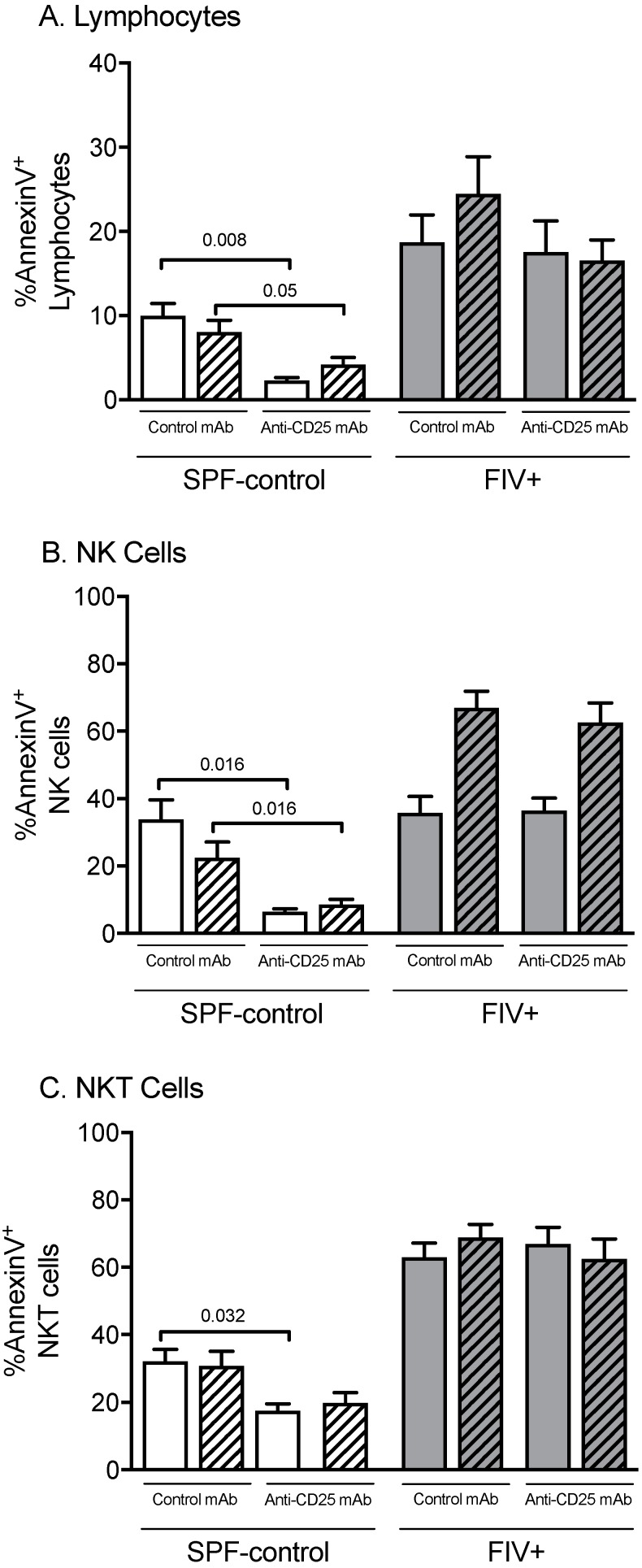
The effects of Treg depletion on total lymphocyte, NK cell, and NKT cell apoptosis. Apoptosis was assessed in cats treated with isotype control mAb or anti-CD25 mAb by Annexin V staining, followed by flow cytometric analysis of lymphocytes from the control lymph node (open bars) versus Lm-draining lymph node (hatched bars). (**A**) Percentage of lymphocytes expressing Annexin V; (**B**) percentage of NK cells expressing Annexin V; (**C**) percent NKT cells expressing Annexin V. Statistical analysis was performed using one-way ANOVA with Tukey’s post-test; *n* = 6 for each treatment group; *p*-values are shown above brackets that indicate group comparisons.

FIV infection leads to increased apoptosis of lymphocytes, as determined by Annexin V expression in both CLN and Lm-LN when compared to SPF-control cats [10]. Treg cell depletion led to a reduction in Annexin V^+^ total lymphocytes, NK cells, and NKT cells in both CLN and Lm-LN of SPF-control cats, but not FIV-infected cats (Figure 4A–C). This suggests that Treg cells may contribute to apoptosis in healthy cats under normal conditions, but other pro-apoptotic factors exist in chronically FIV-infected cats.

### 3.3. The Effect of Treg Depletion on the Functional Response to Lm

**Figure 5:**
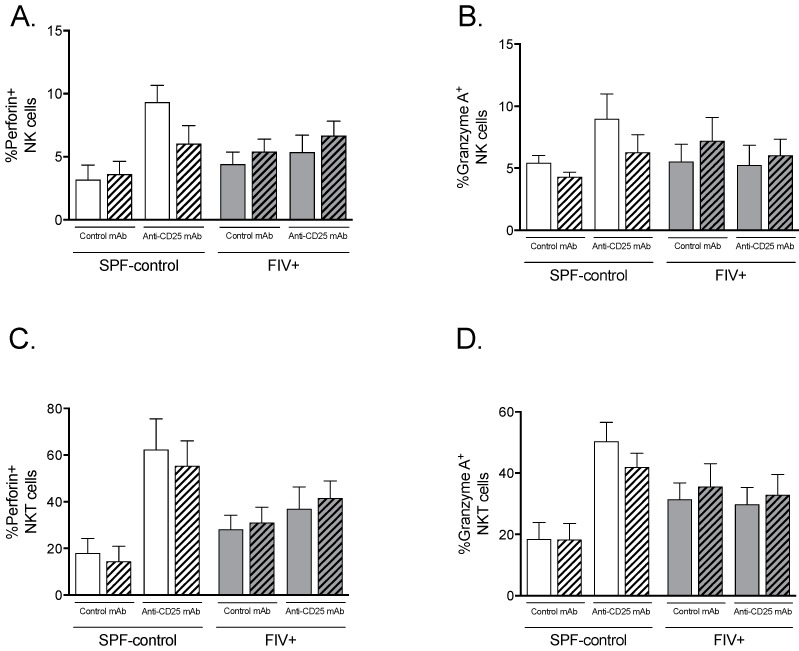
The effect of Treg depletion on the expression of perforin and granzyme A by NK and NKT cells. NK and NKT cell functions were assessed in cats treated with isotype control mAb (open bars) or anti-CD25 mAb (hatched bars) by perforin and granzyme A staining followed by flow cytometric analysis of lymphocytes. Lymph node cells from the control lymph node (open bars) and Lm-draining lymph node (hatched bars) were cultured overnight with IL-2, treated with monensin, stained for lymphocyte subset surface markers and intracellular perforin and granzyme A, then analyzed by flow cytometry. (**A**) Percentages of of total NK cells and (**C**) NKT cells expressing perforin. (**B**) Percentages of NK cells and (**D**) NKT cells expressing granzyme A. Statistical analysis was performed using one-way ANOVA with Tukey’s post-test; *n* = 6 for each treatment group; *p*-values are shown above brackets that indicate group comparisons.

Perforin and granzyme A are functional markers of activated NK and NKT cells that are increased in SPF-control cats challenged with Lm but not in FIV-infected cats [10]. Despite an increase in NK and NKT cell proliferation in response to Lm by FIV-infected cats treated with anti-CD25 mAb, as shown in Figure 3, expression of perforin or granzyme A did not significantly increase in NK cells (Figure 5A,B) or NKT cells (Figure 5C,D).

**Figure 6:**
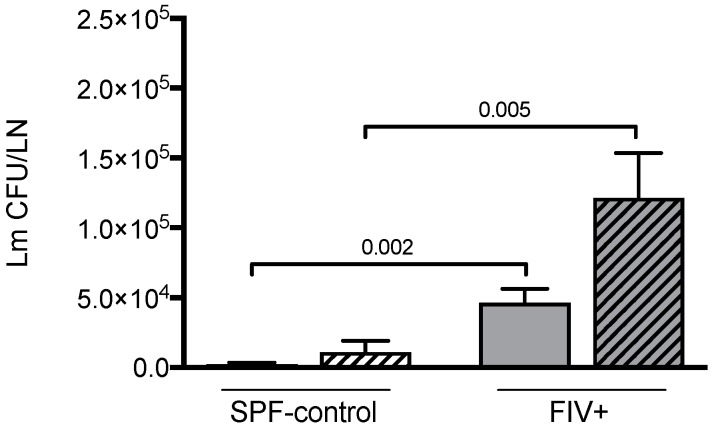
The effect of Treg depletion on Lm clearance. The number of Lm colony-forming units (Lm CFU/LN) were determined 3 days after Lm challenge of SPF-control and chronically FIV-infected (FIV+) cats. Isotype control mAb treated cats (open bars) and anti-CD25 mAb treated cats (hatched bars) are shown. Statistical analysis was performed using Mann–Whitney U test; *n* = 6 for each treatment group; *p*-values are shown above brackets that indicate group comparisons.

We have previously shown that FIV infection impairs the initial control of Lm infection compared to SPF control cats [8,9]. Here we confirm our previous observations and further show that depletion of CD4^+^CD25^+^ cells does not result in more effective clearance of Lm in either FIV-infected or SPF-control animals (Figure 6).

### 3.4. Treg Depletion Does Not Affect Dendritic Cell Percentage or Activation Status

**Figure 7:**
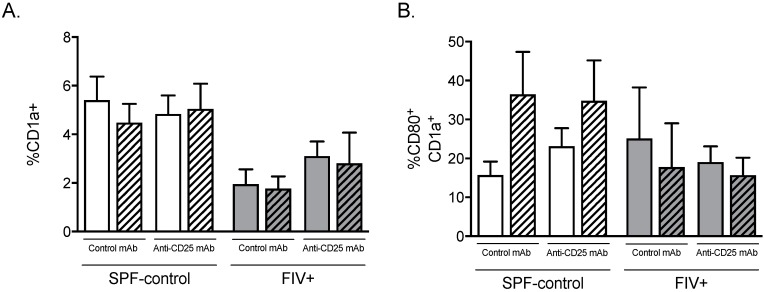
The effect of Treg depletion on dendritic cell (DC) percentage and activation status. Cats were treated with isotype control mAb or anti-CD25 mAb and cells from the control lymph node (open bar) and Lm-draining lymph node (hatched bar) were analyzed by flow cytometric analysis. (**A**) Percentage of CD1a^+^ DC and (**B**) percentage of CD1a^+^ DC expressing CD80. Gating included all nucleated lymph node cells. Statistical analysis was performed using one-way ANOVA with Tukey’s post-test. No significant differences were identified; *n* = 6 for each treatment group.

Humann and Lenz showed that activation of NK cells during Lm infection requires secreted cytokines and ligation of NK activating receptors by dendritic cells [27]. We sought to determine whether Treg cell depletion would alter the number or activation status of CD1a^+^ dendritic cells (DC) in LN. Treg depletion did not increase the percentage of CD1a^+^ DC in FIV-infected cats after Lm challenge (Figure 7A). Likewise, Treg depletion did not affect the percentage of CD1a^+^ DCs expressing the activation co-receptor CD80 (B7.1) (Figure 7B). Overall, frequency of CD1a^+^ DCs was between 1.5 and three-fold higher in the LN of SPF-control cats compared to LN of FIV-infected cats (Figure 7A).

## 4. Discussion

In the present study, we explored the role of Treg on the innate immune response of chronically FIV-infected cats challenged with the opportunistic pathogen *Listeria monocytogenes*. As we have previously reported, treatment with anti-feline CD25 mAb resulted in depletion of Treg cells, as measured by the frequencies of CD4^+^CD25^high^ and CD4^+^CD25^high^FOXP3^+^ cells [7,23]. Importantly, treatment with anti-feline CD25 mAb did not deplete CD25+ NK cells and NKT cells. While treatment of chronically FIV-infected cats with anti-CD25 mAb or isotype control mAb did not affect cell-associated viral burden [7], an increase in plasma viremia three days after Lm challenge was seen, presumably due to viral activation during the inflammatory response to Lm. Such an association of a transient increase in viremia with opportunistic infections is well described during chronic infection with HIV-1 [28].

Generalized immune activation is one of the hallmarks of HIV-1 infection. Heightened lymphocyte proliferation is reported to be one of the consequences of high levels of immune activation during HIV-1 infection [29,30]. A higher constitutive turnover of NK and T cells has been described during both SIV [31,32] and HIV infections [33,34]. We have also reported increased constitutive cell proliferation during chronic FIV infection that does not further increase in response to Lm challenge, suggesting FIV infection not only drives cell proliferation but also renders these cells unresponsive to further stimuli [10]. Treg cells suppress proliferation and function of a wide range of cells, including conventional CD4^+^ and CD8^+^ T lymphocytes [35,36,37], B cells [38], dendritic cells [39], monocytes/macrophages [40], NKT cells [41], and NK cells [42,43]. In the present study, depletion of Treg led to an increase in the total number of cells in the Lm-LN of FIV-infected cats. Treg depletion also resulted in NK and NKT cell proliferation in response to Lm challenge, similar to that observed in SPF-control cats.

Despite an increase in NK and NKT cell numbers after Treg depletion, there was not commensurate improvement in listericidal competence of FIV-infected cats. This suggests Treg depletion did not improve NK and NKT cell function. In contrast to the increase in expression of perforin and granzyme A in NK and NKT cells of SPF cats upon Treg depletion, no such increase was observed in FIV-infected cats. A plausible explanation for this observation could be that NK cells require activation by other cells that were not rescued by Treg cell depletion. Mature DCs prime and activate NK cells through cell-to-cell contact dependent signals and soluble mediators, such as IL-12, IL-15 (membrane-bound and soluble), IL-18, and type I interferons [44,45,46]. Our previous observations show that locally delivered IL-15 can rescue the ability of FIV-infected cats to control initial Lm infection [9] and the present study suggests impaired NK cell function may be due to a defective DC population. Indeed, we observed an overall decrease in the percentage of CD1a^+^ DC in the lymph nodes of FIV-infected cats. SPF-control cats showed an increased percentage of CD1a^+^ DC expressing the costimulatory molecule CD80 (B7.1) after challenge with Lm, whereas upregulation of CD80 was not observed in FIV-infected cats. This suggests an incomplete activation/maturation of DCs in response to Lm, a representative opportunistic pathogen, during chronic FIV infection. These results are consistent with our previous report describing impaired CD1a+ DC migration and co-receptor upregulation after peripheral (subcutaneous) stimulation [47]. Whether there is an underlying deficiency in IL-15 production, possibly due to defective DC function, remains to be determined. During the acute Friend retrovirus infection of mice, Treg suppression of NK cell proliferation and effector functions is associated with Treg consumption of IL-2 and increased production of IL-10 [48,49]. In this acute disease model, depletion of Treg results in increased DC activation and IL-15 levels [48]. Interestingly, treatment of HIV-1 infected individuals with IL-15 has been proposed as a means to stimulate NK cell killing of HIV-infected cells after latent virus reactivation [50].

Consistent with our previous findings, FIV infection is associated with a higher constitutive percentage of cells undergoing apoptosis; however, this was unaffected by Treg cell depletion. In contrast, we observed an overall reduction in apoptosis after Treg depletion in LN cells from SPF-control cats. Thus, under normal conditions, apoptosis appears to be related at least in part to Treg cells, perhaps through the perforin/granzyme pathway [51,52]. Other apoptotic mechanisms associated with FIV/HIV during the chronic phase of infection remain to be investigated.

## 5. Conclusions

In conclusion, there was no improvement in clearance of Lm, a representative opportunistic pathogen, after removal of Treg cells during chronic FIV infection, reducing the likelihood that there is a directly negative effect by Treg cells on NK and NKT cells in vivo. Whether there is an indirect effect of Treg cells on NK and NKT cell functions and/or whether the relationship between these cell types is different during the acute phase of FIV disease is unknown.

## Supplementary Materials

Supplementary materials can be found at https://www.mdpi.com/1999-4915/11/11/984/s1. Supplemental Figure S1. Effect of anti-CD25 mAb treatment on cell-associated viral burden.
