# Supplementary material for: Effects of Regulatory T Cell Depletion on NK Cell Responses against Listeria monocytogenes in Feline Immunodeficiency Virus Infected Cats
Source: Viruses. 2019 Oct 24;11(11):984. doi: 10.3390/v11110984 (PMC6893779; doi:10.3390/v11110984)
Supplement: Supplementary file 1 [file viruses-11-00984-s001.pdf]

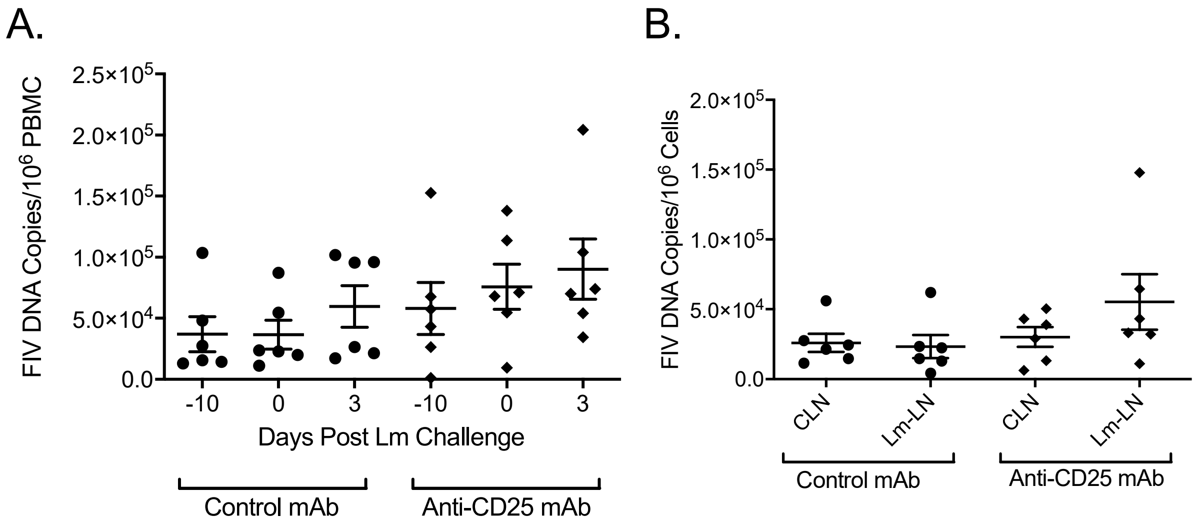

Supplemental Figure 1. Effect of anti-CD25 mAb treatment on cell-associated viral burden. FIV proviral load in peripheral blood mononuclear cells (PBMC)(A.) and lymph node (LN) cells (B.) on days -10, 0 and 3 post-Lm infection. Chronically FIV-infected cats were treated with either control mAb (circles) or anti-CD25 mAb (diamonds). Statistical analysis was performed using 1-way ANOVA with Tukey's post-test. N=6 for each treatment group.
